# Obesity and oxidative stress: potential mechanisms in endometrial disorders

**DOI:** 10.3389/fendo.2026.1709556

**Published:** 2026-02-11

**Authors:** Yiwei Wang, Zhilin Chen, Ruiqing Wang, Aiqun Song, Yangpu Zhang

**Affiliations:** 1 Hubei University of Chinese Medicine, Hubei, Wuhan, China; 2 Hubei Provincial Hospital of Traditional Chinese Medicine, Hubei Wuhan, China; 3 Hubei Shizhen Laboratory, Hubei, Wuhan, China; 4 Affiliated Hospital of Hubei University of Chinese Medicine, Hubei, Wuhan, China; 5 Hubei Provincial Clinical Research Center for Acupuncture and Moxibustion in Obesity Treatment, Hubei, Wuhan, China; 6 Xinhua Hospital of Hubei University of Chinese Medicine, Hubei, Wuhan, China; 7 Hubei Provincial Hospital of Integrated Chinese and Western Medicine, Hubei, Wuhan, China

**Keywords:** endometrial, endometrial dysfunction, endometrial disease, obesity, oxidative stress

## Abstract

Obesity is a systemic metabolic disorder that is inducing factor for other diseases such as diabetes mellitus, cardiovascular diseases, malignancies, hepatic dysfunction, renal dysfunction and endometrial diseases. Emerging evidence has shown that Oxidative stress (OS) plays a key mediator in the development of obesity and its complications. Obesity itself can produce OS through several different pathways, including disrupting energy metabolism, interfering with endocrine homeostasis, inducing systemic chronic inflammatory responses and changing gut microbiota. Among the complications induced by obesity, endometrial diseases have been closely related to OS. OS damages molecular phenotype of endometrial cells, induces endometrial apoptosis and affects endometrial angiogenesis, decidualization and receptivity. In this review, we will summarize the relationship among obesity, OS and endometrium, that is, how obesity can induce OS with various pathways, how OS damage endometrial structure and function, and further explore the relationship between OS and obesity-associated endometrial disorders and the potential of using antioxidant strategy as a new therapeutic method.

## 1 Introduction

Obesity is a chronic metabolic disease with both genetic and environmental aetiologies, and associated metabolic risks can greatly impair the physiological and psychological well-being of individuals. It is estimated that there are more than one billion individuals with obesity in the world and the high prevalence of obesity has emerged as a global public health problem with significant effects on human health (1). Oxidative stress (OS) occurs when there is a breakdown in the dynamic balance between the production and removal of reactive oxygen species (ROS), and excessive ROS can induce DNA, protein and lipid damages and lead to cellular dysfunction (2). Obese women are frequently in a state of OS, which may not only impact on metabolism but also impair the normal structure and function of endometrium and further impair fertility (3). In this review, we will focus on the crosstalk between obesity and OS, and their effects on endometrial physiology and pathology to elucidate the possible mechanisms underlying endometrial disorders.

## 2 Methods

In this narrative overview, research and review papers were obtained using the university E-Library academic search tools PUBMED and WEB OF SCIENCE global search tool, including literatures published in English and available up to July 2025. The following key word were used for the search alone or in combination: Endometrium, Endometria, Obesity, Appetite Depressants, Body Weight, Anti-Obesity Agents, Bariatrics, Metabolism, endocrine, inflammation, gut microbiota, OSes, Oxidative Damages, Oxidative Injury, Anti-OS, Abnormal Uterine Bleeding, Endometrial Polyps, Endometriosis, Endometrial Hyperplasia, Endometrial Cancer, Antioxidants. Literatures were selected for review based on their titles and abstracts which were relevant to the topic. The references of the articles correlating to this review were further searched and selected.

## 3 Obesity, OS and endometrium

**Figure 1:**
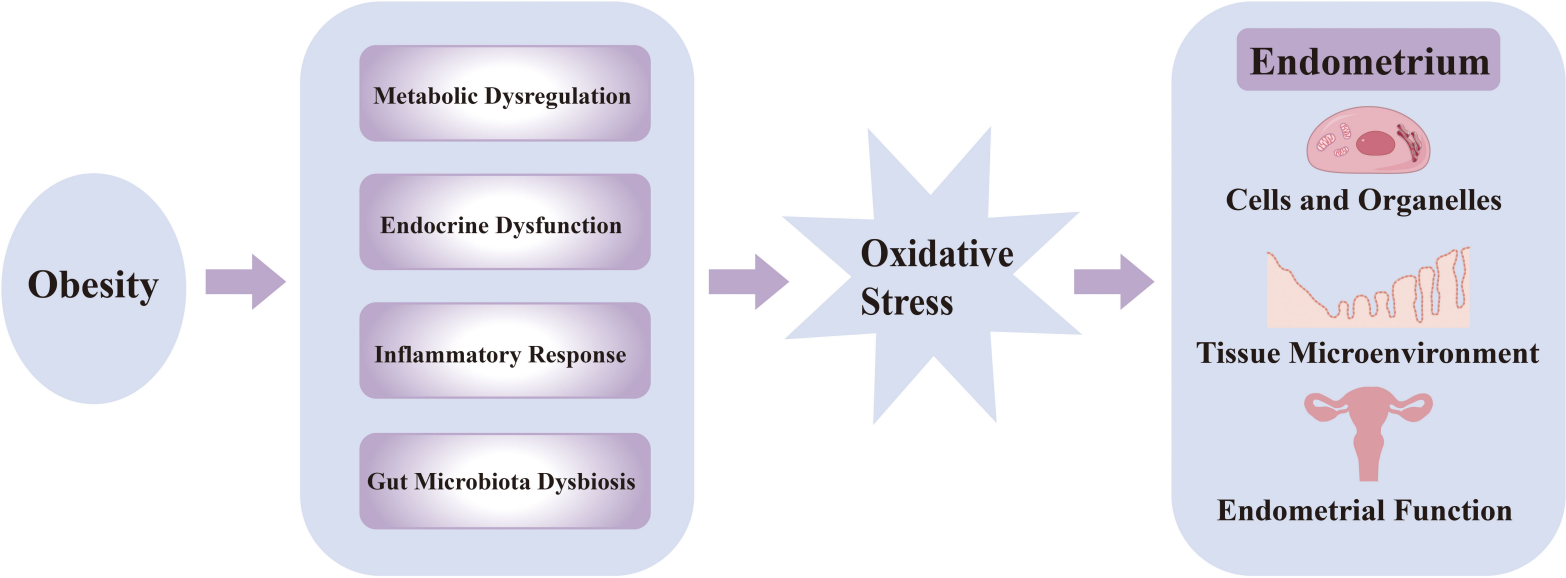
Possible interrelationships among obesity, oxidative stress, and the endometrium.

The balance between the pro-oxidant and antioxidant systems is disrupted, which will lead to the generation of ROS and reactive nitrogen species (RNS). When ROS and RNS exceed the body’s antioxidant defense capacity, OS occurs. Excessive ROS can damage cellular DNA, proteins, and lipids, resulting in cellular dysfunction (4).A complex bidirectional relationship exists between obesity and OS: on one hand, excessive lipid accumulation in obesity induces systemic OS; on the other hand, OS further exacerbates obesity-related metabolic disturbances, creating a vicious cycle. Clinical data from Shigetada Furukawa et al. (4) demonstrate a positive correlation between systemic OS levels and body mass index (BMI), and this finding was also verified in the obese mouse model. OS also impacts the endometrium, promoting structural and functional alterations. While a certain level of OS is involved in regulating physiological endometrial states, excessive OS can adversely affect cellular components, vascularization, epithelial integrity, decidualization, and endometrial receptivity. Obesity exacerbates OS, suggesting that obesity, in conjunction with OS, may represent one of the potential mechanisms contributing to the development and progression of endometrial disorders, shown as **Figure 1**.

## 4 Mechanisms of obesity-induced OS

ROS and RNS are normal products of cellular metabolism and are essential for many physiological cellular functions. However, the ROS production is elevated in adipocytes with fat overload (5). Obesity can cause OS through following pathways: disrupting lipid metabolism, interfering with endocrine function, stimulating inflammatory response, and changing composition of gut microbiota.

### 4.1 Obesity-induced metabolic dysregulation and OS

Excessive adipose tissue accumulation in individuals with obesity leads to a significant increase in plasma free fatty acid (FFA) levels (6).When these FFA are transported to non-fat tissues, they enter the mitochondria for oxidation, disrupting normal fatty acid β-oxidation processes. At the same time, mitochondrial dysfunction in individuals with obesity also causes abnormal fatty acid oxidation, manifested as enhanced mitochondrial β-oxidation and reduced tricarboxylic acid (TCA) cycle activity. This incomplete oxidation process generates excessive ROS, particularly superoxide anions (O_2_^-^), thereby inducing OS (7).

**Figure 2:**
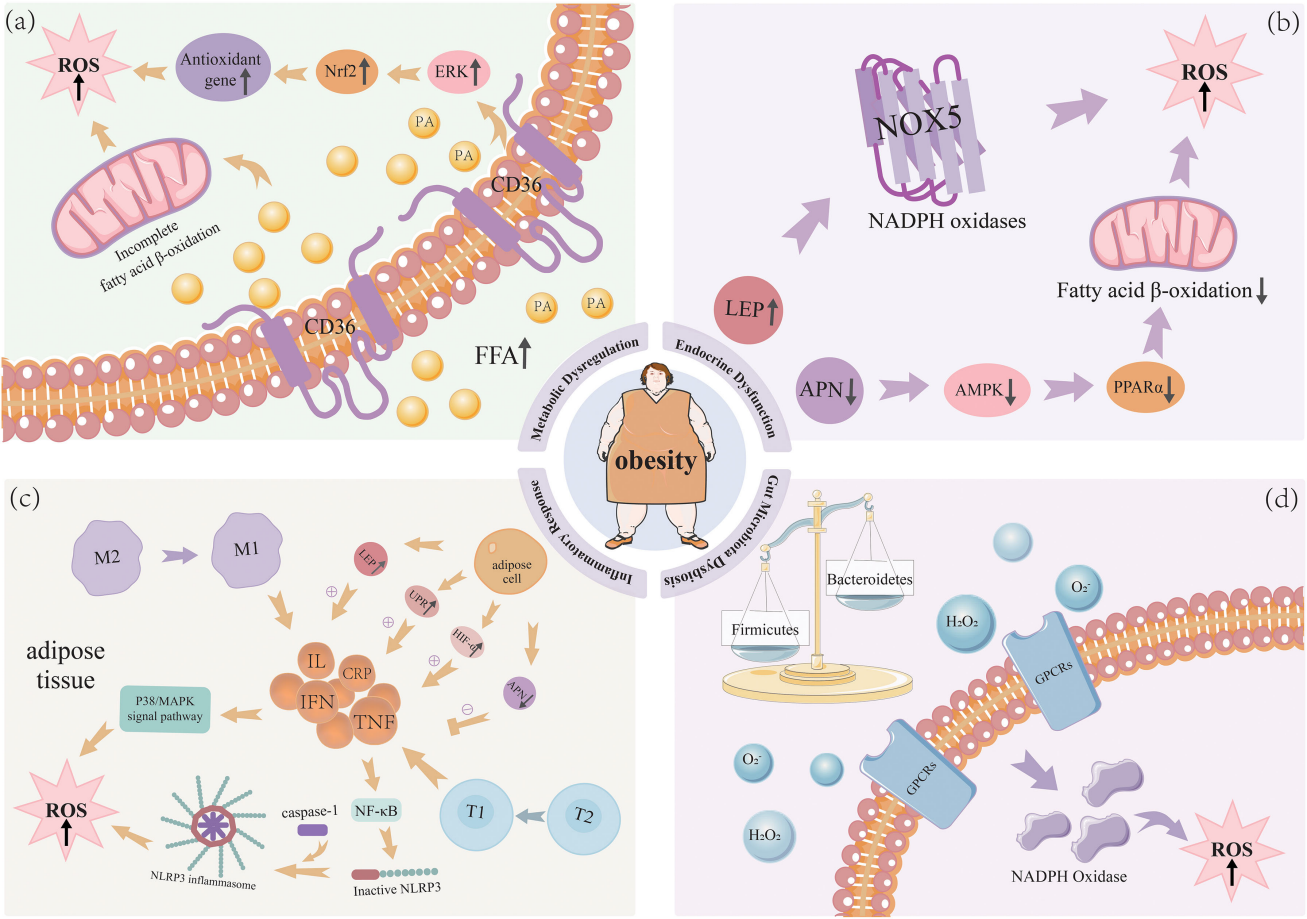
Mechanisms underlying obesity-induced oxidative stress.

Palmitic acid (PA), the most abundant saturated fatty acid in humans accounting for 20-30% of total fatty acids and widely present in diets (8), has been demonstrated to increase harmful lipid production, impair cellular function, and promote OS-related pathologies when excessively taken up by non-adipose tissues (9, 10).Wei Hua et al. treated mouse podocyte cell lines with PA and observed that PA upregulated the expression of fatty acid translocase CD36 in podocytes. PA-treated podocytes showed significantly increased ROS generation, while CD36 inhibition correspondingly reduced ROS levels, suggesting that elevated lipid levels induce CD36-mediated fatty acid uptake and subsequent OS in podocytes, shown as **Figure 2a**.

Shi et al. (11) demonstrate by experimental that PA activates the ERK-Nrf2-HO-1 pathway, thereby stimulating the production of ROS. The cells treated with PA induced a dose-dependent increases in cellular ROS levels along with elevated total extracellular signal-regulated kinase (ERK) and phosphorylated ERK (p-ERK). Nuclear factor erythroid 2-related factor 2(Nrf2), as a downstream target of ERK, becomes activated to regulate antioxidant gene expression in response to OS. However, under obese conditions, this protective mechanism may become dysregulated through excessive activation, promoting lipogenesis while suppressing lipolysis (12).

### 4.2 Obesity-induced endocrine dysfunction and OS

Adipose tissue is involved in not only storing energy but also as an endocrine organ (13).When in an obese state, adipose tissue releases several hormones such as leptin (LEP) and adiponectin (APN) which are normally counter-regulatory to maintain systemic energy homeostasis. Obesity is associated with greatly increased LEP levels and decreased APN expression (14).

LEP is primarily transported in circulation bound to plasma proteins and crosses into the central nervous system (CNS) via diffusion to induce satiety signaling. Under obese conditions, LEP’s regulatory effects on insulin sensitivity become attenuated, contributing to insulin resistance. This insulin resistance further promotes lipolysis in adipose tissue, releasing excessive FFAs that subsequently induce OS (15). Additionally, research has demonstrated that LEP can stimulate intracellular ROS generation through NADPH oxidase activation. Sinda Mahbouli et al (16). confirmed in cellular models that LEP induces ROS production regardless of concentration, with significant upregulation of NADPH oxidase 5 (NOX5) expression observed across all experimental conditions.

In contrast to LEP, the expression of APN is inversely related to the degree of obesity, and usually there is a decreased expression of APN in obese patients (17). APN has strong antioxidant activities to inhibit the generation of ROS and eliminate the oxidative cellular damage (18). It has been reported (19) that APN activates its receptors to subsequently stimulate AMP-activated protein kinase (AMPK) and peroxisome proliferator-activated receptor alpha (PPARα) to enhance the oxidation of fatty acids and improve energy metabolism, and to reduce the accumulation of lipid and OS (20). Therefore, the decreased expression of APN or signaling of APN may be upstream mechanisms for the OS induced by obesity. shown as **Figure 2b**.

### 4.3 Obesity-induced inflammatory response and OS

Obesity is not merely an excess of energy but also a systemic chronic low-grade inflammatory state (21). Adipose tissue in obese individuals secretes multiple pro-inflammatory cytokines, including TNF-α, IFN-γ, and ILs, thereby triggering systemic low-grade inflammation. These inflammatory mediators stimulate macrophages and monocytes to produce ROS and RNS (22). Consequently, elevated concentrations of inflammatory factors may be a key factor contributing to increased OS, shown as **Figure 2c**.

#### 4.3.1 Adipose tissue-derived inflammatory factors

During obesity, as adipose tissue volume increases, localized hypoxia develops. Under this hypoxic and high-fat dietary environment, adipose tissue exhibits significant immune cell infiltration (macrophages, T cells) accompanied by characteristic phenotypic shifts (23): Macrophages polarize from the anti-inflammatory M2 phenotype toward the pro-inflammatory M1 phenotype, while T cells differentiate toward the type 1 helper T cell (Th1) subset rather than the type 2 helper T cell (Th2) subset (24, 25). Notably, endocrine dysregulation in obesity further fuels inflammation. Elevated LEP and APN deficiency enhance Th1 pro-inflammatory effects and M1 macrophage activity (26, 27). These immune cells promote IFN-γ, TNF-α, and IL-12 secretion via the TLR4/NF-κB pathway. These cytokines further drive immune cell phenotype shifts, establishing chronic low-grade inflammation (28). As confirmed by Frank M Schmid’s research (29), levels of IL-5, IL-10, IL-12, IL-13, and IFN-γ are significantly elevated in both generalized and abdominal obesity.

Local hypoxia during adipocyte hypertrophy also induces endoplasmic reticulum stress (ERS) (30). ERS triggers a complex adaptive response known as the unfolded protein response (UPR) (31). The standard UPR comprises three branches mediated by three transmembrane proteins on the ER: protein kinase R-like endoplasmic reticulum kinase (PERK), inositol-requiring enzyme 1 (IRE1), and transcription factor 6 (ATF6). These pathways activate inflammatory signaling, including JNK, IKK, and NF-κB, leading to increased expression of inflammatory cytokines (32, 33).

#### 4.3.2 Cytokine-mediated ROS generation and OS

Inflammatory cytokines promote ROS production through multiple pathways (34). IL-1β activates the p38 mitogen-activated protein kinase (p38/MAPK) pathway, which plays a crucial role in OS and inflammatory responses. The activated p38/MAPK induce neutrophils to release superoxide anions, thereby increasing the generation of ROS. Proinflammatory cytokines (IL-1β, IL-6, TNF-α) further recruit ROS-producing neutrophils, amplifying inflammation (35).

ROS activate the NLRP3 inflammasome (36), a multiprotein complex in innate immunity. NLRP3 activation promotes caspase-1 maturation, which processes pro-IL-1β and pro-IL-18 into active forms (37). Under OS, excessive ROS also trigger release of inflammatory cytokines and transcription factors, establishing a positive feedback loop that perpetuates both OS and inflammation (38).

### 4.4 Obesity-induced gut microbiota dysbiosis and OS

Through extensive studies, it has been demonstrated that obesity markedly changes the composition of microbiota (39), characterized by decreased diversity of microbiota compared with that in normal-weight individuals (40). This decrease in diversity results in the disturbance of the balance between beneficial and pathogenic bacteria, and a relative increase in the abundance of harmful bacterial populations. Specifically, individuals with obesity present an increased Firmicutes-to-Bacteroidetes ratio. Given that the Firmicutes can increase the energy harvest efficiency of dietary materials, individuals with obesity present increased metabolic disturbance (41).

The gut microbiota generates various reactive metabolites (42), including hydrogen peroxide (H_2_O_2_) and superoxide anions (O_2_^-^), which directly modulate intestinal redox homeostasis. When intestinal epithelial cells and immune cells detect bacterial components and metabolites through pattern recognition receptors (e.g., Toll-like receptors, TLRs), they activate NADPH oxidases, resulting in ROS production (O_2_^-^, H_2_O_2_). While these ROS serve antimicrobial functions, excessive generation may cause oxidative damage to host cells, precipitating OS (43).

The intestinal barrier integrity is damaged by enhanced OS. The gut permeability is increased, and the harmful materials (such as endotoxins) are systemically translocated due to the intestinal barrier damage. This results in systemic inflammation and OS (43). Moreover, the gut dysbiosis can induce chronic low-grade inflammation, and the inflammatory environment increases the ROS level, which also establishes the vicious cycle of OS (44), shown as **Figure 2d**.

## 5 Mechanisms of OS-mediated pathological changes in the endometrium

**Figure 3:**
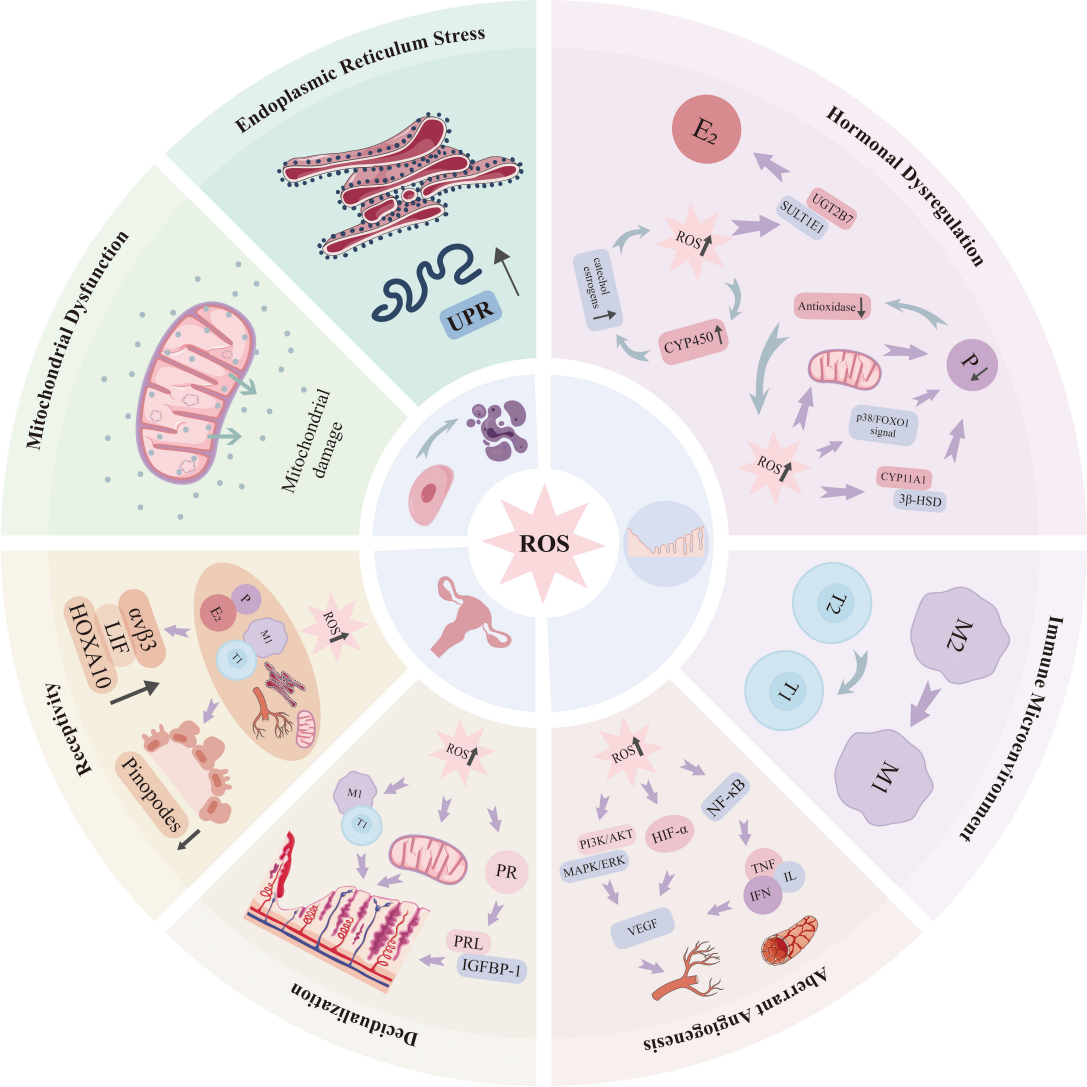
Mechanisms underlying the impact of oxidative stress on endometrial structure and function.

OS may serve as a pivotal link between obesity and endometrial pathology, and is one of the factors promoting the onset and progression of endometrial diseases. Under physiological conditions, moderate levels of ROS maintain endometrial physiological functions. However, when obesity-induced ROS levels exceed the threshold of cellular antioxidant defenses, OS triggers a cascade of reactions—from cell and subcellular organelle damage to microenvironmental disruption—ultimately leading to endometrial dysfunction, as shown in **Figure 3**.

### 5.1 Organelle and cellular-level damage

#### 5.1.1 Mitochondrial dysfunction and apoptosis

OS induces damages to mitochondrial function. With the establishment of H_2_O_2_-induced OS model, Chen Xiunan et al (45). found that the mitochondrial membrane potential of endometrial stromal cells was significantly decreased, and the decrease in mitochondrial membrane potential aggravates the mitochondrial damage. In the investigation of OS-induced injury mechanisms in bovine endometrial epithelial cells (BEECs), Pengjie Song et al (46). found that the high level of ROS and low mitochondrial membrane potential could reduce the function of mitochondria through lowering the Bcl-2/BAX ratio with the increase in ROS level.

Besides displaying OS-induced mitochondrial dysfunction, Pengjie Song et al. (46) also found that Cyto-C and caspase-3 expression were simultaneously upregulated. Caspase-3 is a typical downstream effector of apoptosis (47, 48).Ra Oh et al (49) found that p,p'-DDT exposure enhanced the mRNA expression of caspase-3, -6, and -8 in human endometrial stromal cells under OS and finally induced apoptosis. In addition, p,p'-DDT can induce an imbalance between proliferation and apoptosis in human endometrial stromal cells through the generation of OS, change in estrogen receptor (ER) expression, and modulation of PI3K-AKT/ERK/NF-κB signaling pathways. ROS can induce damage to both nuclear and mitochondrial DNA through multiple mechanisms and also damage the integrity of mitochondrial permeability transition pore (mPTP), thereby aggravating the dysregulation of mitochondrial dynamics and the control of apoptosis (50–52).

#### 5.1.2 Endoplasmic reticulum stress and cellular senescence

Endoplasmic reticulum stress induced by high-fat conditions also intensifies with the onset and progression of OS. Excessive ROS disrupt the oxidative folding environment within the ER, further activating the UPR. Sustained UPR activation not only promotes the production of inflammatory mediators as previously described, multiple studies have also reported its association with cellular senescence (53). In OS-induced senescence models, PERK-mediated upregulation of CCAAT/enhancer-binding protein homolog occurs (54). This PERK activation triggers the Akt/p21 pathway, inducing cellular senescence and diminishing regenerative capacity (55).

### 5.2 Microenvironmental disruption

#### 5.2.1 Immune microenvironment alterations

OS induced by obesity damages endometrial immune microenvironment and immune tolerance (56). Reports have demonstrated that OS could induce phenotypic changes in multiple kinds of endometrial immune cells (57): the enhanced cytotoxicity of natural killer (NK) cells and decreased IFN-γ secretion damage the remodeling of placental blood vessels; the enhanced polarization of M1 macrophages and suppression of FOXP3+ regulatory T cells enhance inflammatory response in individuals with obesity and cause immune tolerance defect (23).

#### 5.2.2 Sexual hormone level disruption

Under normal conditions, estrogen(E_2_) undergoes two successive phases of metabolism: cytochrome P450 enzymes (such as CYP1A1) first metabolize E_2_ into reactive metabolites (2-hydroxyestrogens and 4-hydroxyestrogens), which are then conjugated by sulfotransferase (SULT1E1) and UDP-glucuronosyltransferase (UGT2B7) to add a sulfonate (SO_3_^-^) group and a glucuronide group, respectively (58). SULT1E1 and UGT2B7 catalyze the last two steps of E_2_ metabolism that convert reactive E_2_ metabolites into their respective conjugates, which are water-soluble metabolites that are easily excreted. When ROS are excessively accumulated, the equilibrium of E_2_ metabolism is broken by: one, upregulating the expression of cytochrome P450, leading to the enrichment of redox-active catechol E_2_s, which continually induce ROS generation (59); the other, downregulating the transcriptional and catalytic activities of SULT1E1 and UGT2B7, which weaken the E_2_ conjugation and excretion. The decrease in metabolic capacity causes the accumulation of reactive E_2_ metabolites, which further aggravate metabolic disorder (60).

Progesterone(P) synthesis via multiple pathways, and P itself has antioxidant effects, which may, in certain circumstances, enhance OS and create a vicious cycle. Firstly, P is an important gestagen, mainly secreted by the ovarian corpus luteum in the luteal phase. Its synthesis is highly dependent on mitochondrial integrity and the supply of ATP. When the mitochondrial excessive accumulation of ROS damages the structure of luteal cells and the placental trophoblasts, reduces ATP production, the steroid metabolism is consequently disturbed. In addition, ROS can activate p38 MAPK and FOXO1 pathways, leading to trophoblast apoptosis and downregulation of P synthases expression. Besides, ROS can also directly inhibit the activities of key enzymes such as 3β-HSD and CYP11A1. P reduces the expression of P synthases, which leads to the decline of P level via the two mechanisms above (61, 62). Secondly, P can also enhance ROS production. In an IVF superovulation model, Angelo Cagnacci et al. (61) found that high dose progestins with E_2_ enhanced greatly OS, which means that the hormone combination exerts a dose-dependent effect of pro-oxidant versus antioxidant. In an *in vitro* model with mouse renal arterial endothelial cells, Xiao-Hua Yuan et al. (63) found that when H_2_O_2_ alone existed, it could induce increased GSH synthesis, enhanced GPx activity and upregulation of GCLC/GCLM expression. However, when P was added, it could suppress the above antioxidant responses. It decreased GPx activity and GSH content, downregulated GCLC/GCLM expression and then amplified the oxidative signal and weakened the cellular protection.

Furthermore, OS impairs P clearance by disrupting hepatic P metabolism (64).P is normally cleared from the circulation after it is taken up by the liver where it is metabolised by hydroxylation and conjugation into highly excretable products. Impaired P clearance after ROS-induced damage to hepatocyte mitochondria and microsomes with subsequent impairment of CYP450 enzyme activity and reduced efficiency of P hydroxylation/conjugation may also lead to hormonal metabolic dysfunction.

#### 5.2.3 Aberrant angiogenesis in endometrium

Endometrial angiogenesis in the menstrual cycle is a well-organized process where physiological ROS facilitate vascular formation and maturation while supraphysiological ROS concentrations induce pathological processes leading to angiogenic dysfunction (65).

A large amount of evidence has shown that the expression of Vascular endothelial growth factor (VEGF) is regulated by the level of ROS (66, 67), which varies cyclically during the menstrual cycle. Umberto Cornelli et al (68). found that the level of OS increased from menstrual day 1 and reached a peak around day 15 (late proliferative/early secretory phase) and then moderately decreased, which was coincident with significantly increased vascular density in the late secretory phase. This confirmed the positive cross talk between ROS and angiogenesis (69, 70). Moderate ROS can activate PI3K/AKT and MAPK/ERK pathways to upregulate VEGF expression and VEGFR-2 phosphorylation, which further promote endothelial proliferation, migration, and vasculogenesis (71). In addition, menstrual hypoxia can upregulate VEGF through HIF-1α (72). Jiao Cheng (73) found that melatonin could suppress OS-induced HIF-1α/ROS/VEGF signaling and inhibit the proliferation of endothelial cells—indicating that the activation of HIF-1α is an important angiogenic process.

High ROS induce NF-κB signaling activation, up-regulate inflammatory cytokines (TNF-α, IL-1β) which promote inflammation, and further induce the abnormal expression of VEGF, induce abnormal angiogenesis (74). Amalesh Nanda et al (75). found that serum cytokines and VEGF were up-regulated in endometriosis patients. It is speculated that these changes may originate from the activation of NF-κB induced by OS, which can promote vascular hyperplasia and extracellular matrix degradation. Aberrant expression of HIF-1α induced by high OS also participates in the pathogenesis of endometrium (76). As mentioned above, the damage caused by ROS-induced apoptosis further disrupts the vascular structure, and these detrimental effects of aberrant angiogenesis and endometrial diseases progression form a vicious circle.

### 5.3 Endometrial dysfunction

#### 5.3.1Endometrial decidualization

Endometrial decidualization is a process by which endometrial stromal cells transform into decidual cells under the influence of hormones such as P, and it is a prerequisite process for embryo implantation and maintenance of pregnancy. OS induced by obesity disturbs endometrial decidualization through multiple mechanisms. At the signaling pathway level, ROS down-regulate PR signaling through NF-κB/MAPK signaling pathway, and then inhibit the transcription of PRL and IGFBP-1 (77, 78). It has been verified that in obese women and high fat diet mice, the mRNA and protein levels of these key markers of endometrial decidualization (PRL and IGFBP-1) were significantly decreased (79). From the point of view of energy metabolism, some obese patients also present with IR, and hyperinsulinemia + IR further impair endometrial decidualization through suppressing the expression of decidualization-related genes. That is, low expression of SLC2A4 in endometrium impairs the uptake of glucose, and then reduces the efficiency of glycolysis and mitochondrial oxidative phosphorylation. Insufficient ATP and NADPH production further impair normal decidualization (78). In addition, from the point of view of immune regulation, obesity-induced OS can enhance M1 macrophage polarization, increase the cytotoxicity of NK cells, increase the expression of pro-inflammatory cytokines and further impair endometrial decidualization by suppressing the population of FOXP3+ regulatory T cells (24, 25).

Furthermore, decidualization is a process of coordinated changes in cell morphology and function, angiogenesis, extracellular matrix remodeling, and adaptation of the immune microenvironment. OS not only directly damages endometrial decidualization, but also indirectly damages endometrial decidualization through the indirect mechanisms mentioned above, including endometrial cellular dysfunction, abnormal angiogenesis, and disturbance of hormonal metabolism.

#### 5.3.2 Endometrial receptivity

Endometrial receptivity refers to the temporary state in which the endometrium possesses the capacity to support embryo positioning, adhesion, penetration, and implantation. Optimal endometrial receptivity is crucial for achieving successful implantation and maintaining pregnancy (80). Currently identified receptivity molecular markers include adhesion molecules, cytokines, growth factors, and lipids (81). Under OS conditions, influenced by factors such as progesterone resistance, inflammatory interference, and altered immune microcirculation, the expression of receptivity markers like integrin αvβ3, leukemia inhibitory factor (LIF), and the homeobox gene HOXA10 is significantly upregulated. Simultaneously, scanning electron microscopy reveal that under high OS, damage to the actin cytoskeleton impedes microvillus fusion into large pinocytotic protrusions. This results in pinocytotic protrusions exhibiting developmental abnormalities, reduced numbers, or delayed appearance, suggesting diminished endometrial receptivity (82, 83). Ultimately, these combined molecular and morphological alterations cause pathological displacement or closure of the “implantation window,” preventing embryos from achieving normal positioning and adhesion within the endometrium. This represents the primary pathological basis for the low implantation rates and high miscarriage rates observed in obese women.

## 6 Role of OS in obesity-related endometrial disorders

Obesity-induced OS not only causes damage at the molecular level but is also associated with multiple endometrial diseases. In some disorders, OS has been established as an important pathogenic mechanism; while in others, the direct impact of obesity-induced OS has not been established and needs to be investigated.

### 6.1 Abnormal uterine bleeding

AUB refers to any irregular uterine bleeding, including changes in rhythm, frequency, duration, or volume, persisting for six months or longer in non-pregnant women (84). Obesity is a significant risk factor for AUB, particularly ovulatory dysfunction bleeding (AUB-O). A clinical study of 500 women of reproductive age found that 64.2% of those with AUB were overweight or obese (85). Jane J Reavey et al. (86) demonstrated via mouse models that weight gain from high-fat diets impairs endometrial function during menstruation, increasing menstrual blood flow. In guinea pig uterine models treated with combined estrogen and progesterone, elevated OS markers—8-isoprostane and 8-hydroxy-2’-deoxyguanosine (8-OHdG)—occurred concurrently with abnormal vascular morphology (87). Excessive ROS damage to vascular endothelial cells disrupts the balance between vasoconstrictive and vasodilatory factors, impairing the hemostatic contractility of spiral arteries. This results in thin vascular walls and basement membrane defects, promoting irregular uterine bleeding.

### 6.2 Endometrial polyps

EP represent benign lesions characterized by focal overgrowth of the endometrium, closely associated with obesity-related microenvironmental alterations. Studies indicate that women with a BMI ≥ 30 kg/m² exhibit significantly higher polyp incidence compared to normal-weight individuals (88). OS may play a role in this process by regulating key molecular expression (89, 90). The development of EP primarily stems from hormonal imbalances, manifesting as excessive vascular proliferation and abnormal cellular growth. Upregulation of the anti-apoptotic protein Bcl-2 and suppression of the pro-apoptotic protein Bax constitute a crucial mechanism in endometrial polyp development. This apoptotic resistance allows local glandular and stromal cells to evade normal cyclical clearance, thereby forming neoplasms (91, 92). The accumulation of ROS can generate and promote this apoptotic equilibrium. Furthermore, OS induces pathological angiogenesis via the HIF-1α/VEGF pathway, providing essential blood supply support for sustained polyp growth (93). Finally, alterations in the immune microenvironment also contribute to the process. Polyps harbor increased numbers of activated mast cells, which release substantial ROS and inflammatory mediators. Combined with secretory factors (SASP) from senescent cells induced locally by mitochondrial damage or endoplasmic reticulum stress, these elements collectively establish a pro-proliferative, anti-apoptotic inflammatory microenvironment. Thus, EP can essentially be regarded as a pathological overadaptation of systemic obesity-induced OS localized to the uterus.

### 6.3 Endometriosis

EMS is a pathological condition characterized by the presence of functional endometrioid glands and stroma outside the uterine cavity (94). Although benign in nature, EMS exhibits invasive and implanting characteristics similar to malignant tumors. Both obesity and endometrioid lesions are inflammatory diseases, sharing systemic inflammatory features; Paradoxically, studies indicate an inverse dose-response relationship between BMI and EMS prevalence—higher body weight correlates with lower disease incidence (95). Current research unanimously recognizes OS as a pivotal factor. Multiple studies have confirmed the presence of OS biomarkers in patient serum, peritoneal fluid, follicular fluid, ovarian cortex, and both normal and ectopic endometrial tissue (96, 97). ROS catalyzes the progression of EMS across all stages. Menstrual blood, rich in iron ions, generates substantial hydroxyl radicals (·OH) via the Fenton reaction upon retrograde flow into the peritoneal cavity. This directly oxidatively damages peritoneal mesothelial cells, creating conditions for ectopic implantation of endometrial cells. Concurrently, the accumulation of peroxidation products activates the Ras/Raf/MEK/ERK pathway in endometrial cells, upregulating matrix metalloproteinase (MMP-2/MMP-9) expression and causing excessive degradation of the extracellular matrix (ECM). Furthermore, ROS-induced phenotypic changes in macrophages suppress immune function, enabling ectopic endometrial cells to evade immune clearance. Pathological angiogenesis induced by ROS also supplies blood flow to ectopic lesions (96, 98). Thus, this highly oxidative and inflammatory peritoneal environment not only sustains the growth of ectopic lesions but also constitutes the primary cause of patient pain and infertility.

### 6.4 Endometrial hyperplasia

EH, particularly atypical hyperplasia, serves as a precursor lesion for endometrial cancer. Obesity has been identified as a major risk factor for endometrial cancer and its precursor lesion—EH (98). Obesity produces changes in how endometrial cells maintain balance through changes involving oxygen reactions in pathways that process estrogen, and this results in EH and supports movement from the simple form of the condition to the atypical form. As description indicates in previous work, under conditions with high levels of reactive oxygen molecules, estrogens undergo processing by specific enzymes such as CYP1B1 into forms called 4-hydroxyestrogens that show high reactivity, and these then undergo further oxygen reactions into molecules called quinones. These quinones combine with DNA to form structures that link with deoxy purine components, and this results in instability in the genome. More important, stress from oxygen reactions can produce mutations or loss of function in the PTEN gene that suppresses tumors even in the early phases of the condition showing increased cell numbers. Loss of PTEN removes the control that limits the PI3K/AKT pathway that regulates cell growth, and this allows glands in the endometrium to show dense arrangement and nuclear features indicating atypia under continued stimulation by estrogen (99). This shows that stress from oxygen reactions changes the simple disruption of hormones that obesity produces into changes showing increased cell numbers with potential to damage genetic material. This change also provides one mechanism that underlies development of carcinoma in the endometrium.

### 6.5 Endometrial cancer

Obesity is a significant risk factor for endometrial cancer in women. Epidemiological evidence indicates that obesity is the strongest independent risk factor for type I endometrial cancer (100). A meta-analysis of 26 studies by the American Institute for Cancer Research shows that for every 5 kg/m² increase in BMI, the risk of endometrial cancer rises by 50% (101). Although the specific pathogenesis of endometrial cancer remains incompletely understood, numerous studies suggest a link between the disease and OS. Prolonged oxidative exposure leads to substantial accumulation of DNA oxidative damage in endometrial cells, with elevated 8-OHdG levels positively correlated with cancer grade (102). This sustained genotoxic stress depletes the mismatch repair (MMR) system, resulting in microsatellite instability (MSI) phenotypes and conferring a high mutation burden on cells (103). With PTEN function completely lost due to oxidative modification, the PI3K/AKT/mTOR signaling axis becomes persistently activated (104). This not only drives the unlimited proliferation of tumor cells but also confers tolerance to high levels of ROS by upregulating antioxidant systems (such as the Nrf2 pathway), enabling cancer cells to survive and invade in adverse environments (65). Thus, endometrial carcinoma may represent the outcome of obesity-induced OS progressing from quantitative changes (accumulation of DNA damage) to qualitative changes (malignant clonal expansion).

## 7 Therapeutic strategies targeting OS:current status, challenges, and prospects

### 7.1 Current status

Currently, both obesity and endometrial diseases are primarily treated through specialized approaches. Lifestyle management is employed for obesity, with some patients considering complementary therapies such as traditional Chinese acupuncture. A small proportion of severely obese individuals may undergo pharmacological or surgical interventions. Endometrial diseases are managed through disease-specific interventions, including hormone therapy, anti-inflammatory treatments, and surgery. Clinically, obesity-related endometrial diseases—or the comorbid relationship between obesity and endometrial disorders—often focus on treating the endometrial disease with medication alongside weight loss alone. However, obesity-induced chronic inflammation and OS limit treatment efficacy. Simultaneously, obese patients often experience poor weight loss outcomes due to disease-related stress and multifactorial challenges. In this context, given the role of OS in both obesity and endometrial disorders, managing OS may represent a highly promising therapeutic strategy.

### 7.2 Challenges

Antioxidants demonstrate unique potential in improving obesity and treating endometrial pathologies. For instance, melatonin’s antioxidant and anti-inflammatory properties, along with its role as a metabolic regulator, hold therapeutic value in obesity management (105, 106). Antioxidant vitamins used for endometriosis effectively reduce the severity of dysmenorrhea and improve pelvic pain (107). However, despite strong epidemiological and mechanistic evidence linking obesity-induced OS to endometrial diseases, clinical direct evidence remains insufficient and its clinical application is relatively limited. Current understanding is primarily based on animal models, making it imperative to validate local endometrial OS biomarkers through clinical models. Similarly, while relevant basic research evidence exists, large-scale, multicenter clinical studies are lacking to validate its long-term efficacy.

Antioxidants discussed in recent years regarding obesity and endometrial diseases include vitamins, Omega-3 Fatty Acids, melatonin, N-acetylcysteine, curcumin, etc (108). Vitamins C and E synergistically eliminate ROS in the peritoneal cavity, effectively alleviating endometriosis-related pelvic pain (109); Omega-3 fatty acids reduce pro-inflammatory prostaglandin synthesis by inhibiting COX-2 activity, thereby improving the local metabolic inflammatory microenvironment (110); Melatonin, with its exceptional mitochondrial permeability, directly scavenges ROS and suppresses lesion invasion (111); N-acetylcysteine (NAC), as a glutathione precursor, demonstrates clinically significant reduction in ovarian cyst volume and decreased cellular proliferation activity (112); while curcumin exerts potent anti-inflammatory, antiproliferative, and anti-angiogenic effects by blocking the NF-κB pathway and downregulating VEGF expression (113). In recent research, Xiudan Zheng et al (114). also reported an injectable, biodegradable hydrogel with antioxidant properties composed of thiolated hyaluronic acid (tHA) and thiolated chitosan (tChi). This hydrogel’s antioxidant capacity improves the oxidative microenvironment of damaged uteri, promotes endometrial tissue regeneration, and has been validated in mouse models with thin endometrium. Although antioxidant drugs have become more abundant in recent years, certain issues persist. Early non-specific antioxidants (such as high-dose vitamins) demonstrated highly inconsistent clinical benefits across different studies (115). Simultaneously, drug side effects cannot be overlooked. Resveratrol is considered to have potential therapeutic effects in improving ovarian function, but its teratogenicity has not been ruled out. Current research suggests avoiding resveratrol use during the luteal phase and pregnancy (116). Similarly, the related side effects of other antioxidants have not yet been fully elucidated.

### 7.3 Prospects

Therefore, given the current situation and the challenges of insufficient clinical research, inconsistent efficacy, and unclear side effects, it is proposed that future efforts should focus on conducting large-scale, multicenter clinical studies to validate long-term efficacy, as well as research on drug side effects and dosage optimization. Simultaneously, focus should be placed on precision medicine and the development of targeted therapies (e.g., antioxidants targeting mitochondria), or on establishing personalized treatment plans based on specific OS biomarkers (such as 8-OHdG and MDA levels) in patient circulation or tissues. This approach aims to break the vicious cycle linking obesity to endometrial diseases through antioxidant therapy.

## 8 Conclusion and future perspectives

This review elucidates how obesity induces OS through metabolic, endocrine, inflammatory, and gut microbiota pathways. Subsequently, OS causes further damage at cellular and subcellular levels, within the tissue microenvironment, and in endometrial function, thereby promoting the onset and progression of endometrial diseases. Collectively, these findings demonstrate that OS plays a crucial role in the development of obesity-related endometrial pathology.

However, despite molecular evidence of oxidative damage, antioxidant therapies remain underutilized in the clinical management of obesity-related endometrial disorders. High-quality clinical trials evaluating the efficacy and safety of OS interventions for obesity and endometrial diseases are lacking. Furthermore, areas such as drug selection, adverse effects, optimal dosing, and strategies for targeted antioxidant therapy require further investigation.
