# Supplementary material for: Obesity and oxidative stress: potential mechanisms in endometrial disorders
Source: Front Endocrinol (Lausanne). 2026 Feb 11;17:1709556. doi: 10.3389/fendo.2026.1709556 (PMC12932235; doi:10.3389/fendo.2026.1709556)
Supplement: Supplementary file 1 [file DataSheet1.docx]

Supplementary Material

##
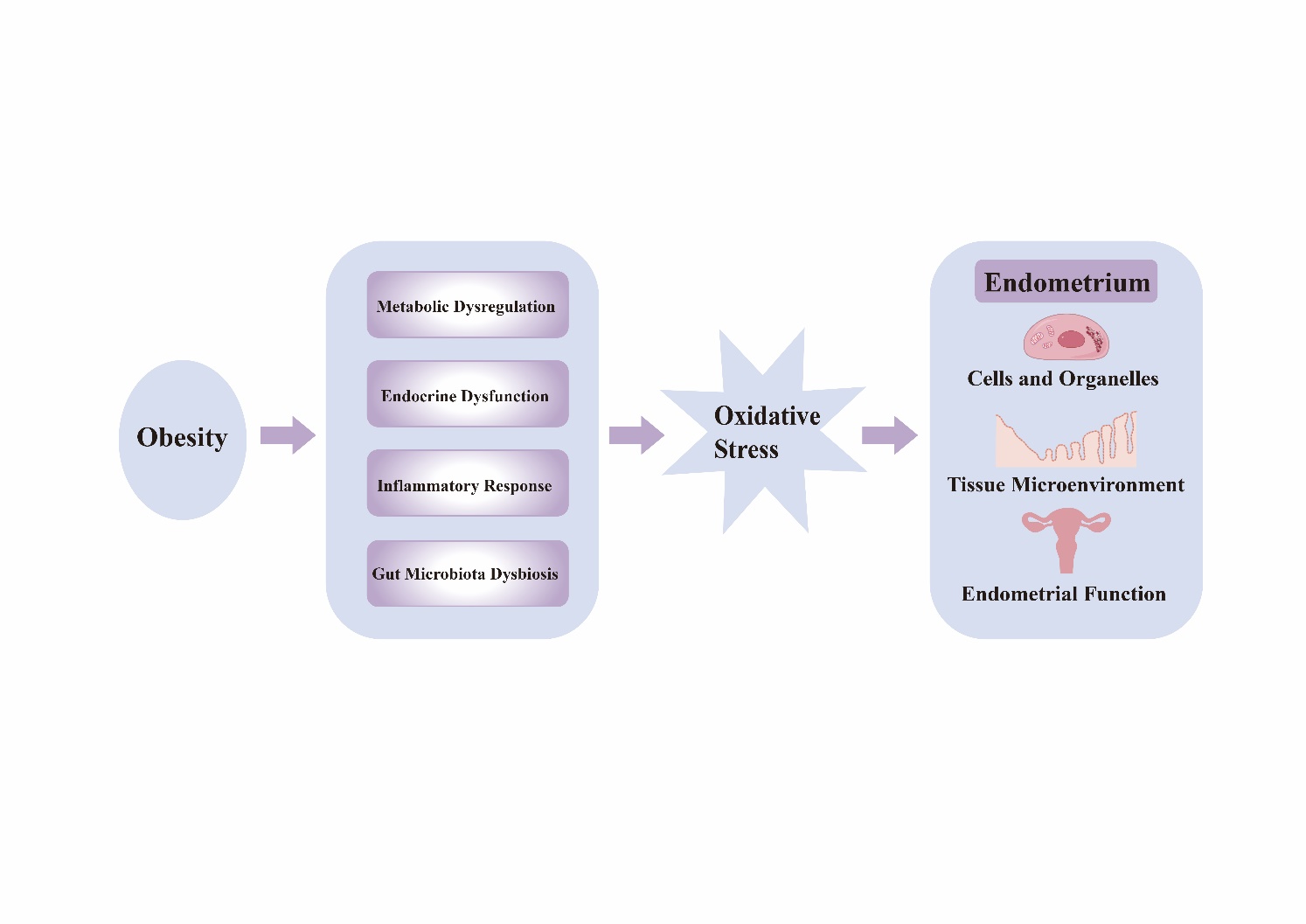
Supplementary Figures

**Supplementary Figure 1.**Possible Interrelationships Among Obesity, Oxidative Stress, and the Endometrium


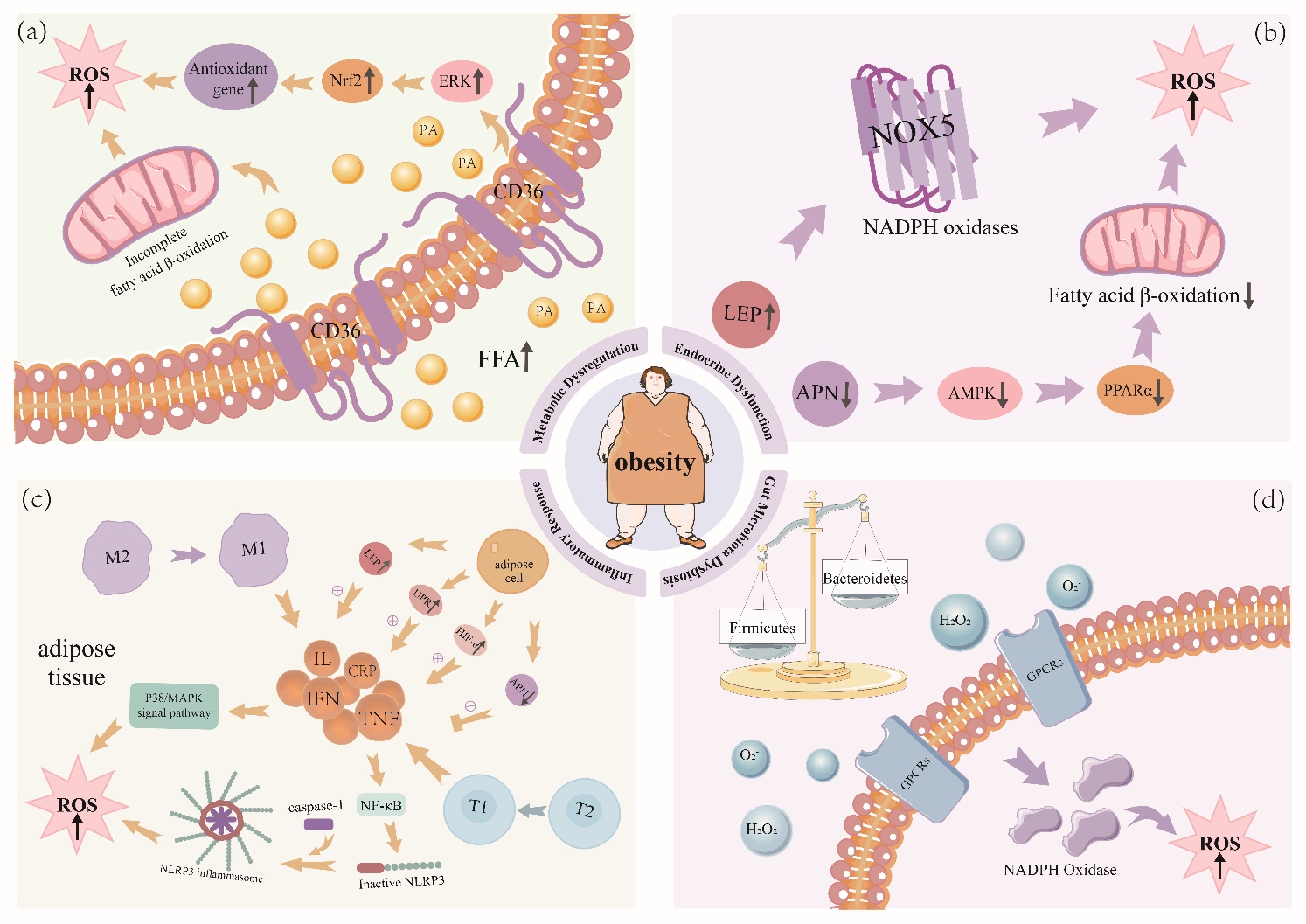


**Supplementary Figure 2.** Mechanisms Underlying Obesity-Induced Oxidative Stress

(a) In obesity, elevated plasma free fatty acids (FFAs) upregulate CD36 expression, promoting FFA transport. Excessive FFAs disrupt normal mitochondrial fatty acid β-oxidation, leading to reactive oxygen species (ROS) generation. Additionally, palmitic acid, one of the FFAs, hyperactivates the ERK–Nrf2–HO-1 pathway, collectively contributing to oxidative stress (OS). FFA, free fatty acid; PA, palmitic acid; CD36, fatty acid translocase; ERK, extracellular signal-regulated kinase; Nrf2, nuclear factor erythroid 2–related factor 2; ROS, oxygen species.

(b) In obesity, increased leptin (LEP) and decreased adiponectin (ADP) levels are observed. Elevated LEP activates NADPH oxidase 5, promoting ROS production. Reduced ADP attenuates the activation of AMP-activated protein kinase and peroxisome proliferator-activated receptor alpha, impairing antioxidant capacity and exacerbating OS. LEP, leptin; ADP, adiponectin; NOX5, NADPH oxidase 5; AMPK, AMP-activated protein kinase; PPARα, peroxisome proliferator-activated receptor alpha.

(c) Obesity promotes a shift in macrophage polarization from M2 to M1 phenotype and a deviation of T cells from Th2 to Th1. Concurrently, hypoxia-inducible factor-1α, the unfolded protein response, and imbalances in LEP and ADP collectively stimulate adipose tissue to release pro-inflammatory cytokines, including tumor necrosis factor , interferon, interleukin, and C-reactive protein. These cytokines activate the p38 mitogen-activated protein kinase signaling pathway and the NLRP3 inflammasome, ultimately inducing OS. HIF-1α, hypoxia-inducible factor-1α; UPR, unfolded protein response; TNF, tumor necrosis factor; IFN, interferon; IL, interleukin; CRP, C-reactive protein; p38/MAPK, p38 mitogen-activated protein kinase.


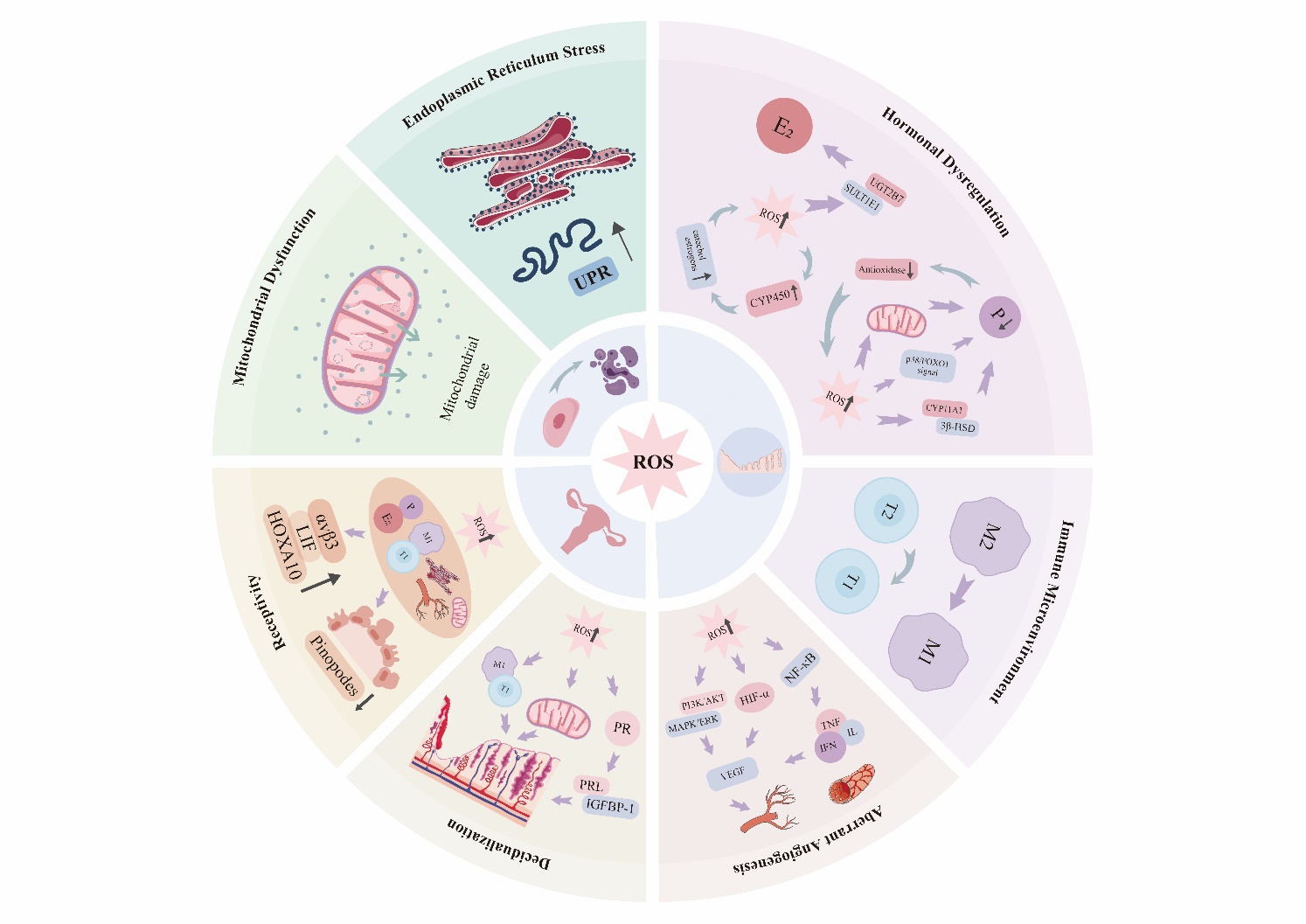
(d) In obesity, gut microbiota-derived metabolites, such as hydrogen peroxide and superoxide anions, activate NADPH oxidase via recognition by G protein–coupled receptors, resulting in ROS production and OS. H₂O₂, hydrogen peroxide; O₂⁻, superoxide anion; GPCRs, G protein–coupled receptors.

**Supplementary Figure 3.** Mechanisms Underlying the Impact of Oxidative Stress on Endometrial Structure and Function.

UPR, unfolded protein response; CYP450, cytochrome P450; SULT1E1, sulfotransferase family 1E, estrogen-preferring, member 1; UGT2B7, UDP-glucuronosyltransferase 2B7; 3β-HSD, 3β-hydroxysteroid dehydrogenase; CYP11A1, cytochrome P450 family 11 subfamily A member 1; P, Progesterone; ER, estrogen receptor; Cyto-C, cytochrome c; VEGF, vascular endothelial growth factor; NF-κB, Nuclear Factor-κappa B; HIF-1α, hypoxia-inducible factor-1α; TNF, tumor necrosis factor; IFN, interferon; IL, interleukin; PR, P receptor; PRL, prolactin; IGFBP-1, factor-binding protein-1; LIF, leukemia inhibitory factor.
